# An update on glucose-6-phosphate dehydrogenase deficiency in children from Brazzaville, Republic of Congo

**DOI:** 10.1186/s12936-019-2688-z

**Published:** 2019-02-28

**Authors:** Nerly Shirère Gampio Gueye, Simon Marie Peko, David Nderu, Felix Koukouikila-Koussounda, Christevy Vouvoungui, Simon Charles Kobawila, Thirumalaisamy P. Velavan, Francine Ntoumi

**Affiliations:** 1 Fondation Congolaise pour la Recherche Médicale (FCRM), Brazzaville, Republic of Congo; 2 Marien Ngouabi University, Brazzaville, Republic of Congo; 3 Institute of Tropical Medicine, University of Tübingen, Tübingen, Germany; 4 Faculty of Medicine, Duy Tan University, Da Nang, Vietnam

**Keywords:** Glucose-6-phosphate dehydrogenase deficiency, Uncomplicated malaria, Republic of Congo

## Abstract

**Background:**

Malaria transmission-blocking anti-malarial drugs, such as primaquine, offers an effective strategy for reducing the incidence of falciparum malaria. However, this drug induces haemolytic anaemia among glucose-6-phosphate dehydrogenase (G6PD) deficient individuals. The distribution of G6PD deficiency in Brazzaville, Republic of Congo and the association of G6PD deficiency with haemoglobin levels and blood cell counts were investigated.

**Methods:**

A total of 212 febrile children were recruited for this study. *Plasmodium falciparum* diagnosis was conducted by microscopy and nested PCR. Sanger sequencing was used to assess G6PD deficiency by detecting 202G>A (rs1050828) and 376A>G (rs1050829) single nucleotide polymorphisms.

**Results:**

Two hundred and twelve children were successfully genotyped for G6PD variants. Overall, 13% (27/212) of the children were G6PD deficient and 25% (25/100) females were heterozygous (11 BA− and 14 A+A−). The remaining 160 children had a normal G6PD genotype. The mean red blood and mean platelet counts were significantly lower in hemizygous male (G6PD A−) participants than in normal male (G6PD A+ or B) participants (*p* < 0.05).

**Conclusion:**

This study gives an update on G6PD deficiency among Congolese children. Understanding the distribution of G6PD deficiency in other geographical regions is recommended before primaquine is adopted in the malaria control programme.

## Background

Glucose-6-phosphate dehydrogenase (G6PD) is the first enzyme of the pentose phosphate pathway [1] and the sole source of reduced nicotinamide adenine dinucleotide phosphate (NAPDH) required by the red blood cells to withstand oxidative stress [2]. It is encoded by a highly polymorphic gene located on the X-chromosome. Over 400 G6PD single nucleotides polymorphism (SNPs) have been identified until 2012 with 186 SNPs being associated with the loss of G6PD activity and stability. The SNPs cause G6PD deficiency which clinically manifests as neonatal jaundice, acute haemolytic anaemia and chronic haemolytic anaemia [3]. However, most people carrying this genetic defect are asymptomatic. Qualitative and enzyme activity tests are currently used to phenotypically classify G6PD deficiency at the point-of-care. Since G6PD deficiency is X-linked, males are classified as either G6PD normal or deficient whereas females are classified into three G6PD phenotypes, normal, intermediate and deficient [4].

The geographical distribution of the various G6PD variants, as defined by the G6PD SNP (s) present, varies extensively. The most prevalent variants include G6PD B (normal), G6PD A+ (moderately deficient) and G6PD A− (severely deficient). Of this, G6PD B, the wild-type variant, is the most common variant globally [5]. G6PD A+ and A− are predominantly found in people of African origin [6, 7]. G6PD A+ is a non-deficient variant arising from 376A>G (rs1050829) point mutation whereas G6PD A−, the deficient variant, arises from two G6PD point mutations, namely 376A>G and 202G>A (rs1050828) [8]. It is estimated that over 400 million people are G6PD deficient globally; making G6PD deficiency the most common human enzymopathy [9].

The prevalence of G6PD deficiency is highly heterogeneous with the prevalence in the tropics ranging from for 3% to 35% [4]. Globally, the highest prevalence of G6PD deficiency occurs in Sub-Saharan Africa where malaria is also prevalent [3]. Due to this overlap, it has been suggested that G6PD deficiency may have arisen from natural selection by malaria. This hypothesis is supported by studies demonstrating a negative association between G6PD A− variant and severe malaria, particularly in hemizygote deficient males and homozygote deficient females [10, 11]. However, a recent systematic review and meta-analysis of published data from East and West Africa revealed the absence of this association [12]. This study also demonstrated that G6PD deficiency may be protective against *Plasmodium falciparum* uncomplicated malaria in Africa [12].

Even though G6PD deficiency is largely asymptomatic, acute haemolytic anaemia can be triggered among G6PD deficient individuals by an infection, ingestion of fava beans or certain drugs [3, 13]. One of the drugs known to induce acute haemolytic anaemia is primaquine [3, 14]. Primaquine (PQ) is an 8-aminoquinoline anti-malarial drug recommended for preventing *Plasmodium vivax* and *Plasmodium ovale* relapse provided the G6PD status is known with exception of pregnant women, children < 6 months years and women breastfeeding children below 6 years old [15].

In the context of falciparum malaria control, a single dose of PQ in combination with artemisinin-based combination therapy (ACT) is recommended for reducing *P. falciparum* transmission to the vectors [13, 15]. High efficacy of PQ against *P. falciparum* gametocytes offers an attractive strategy for reducing malaria transmission in the Republic of Congo and other malaria-endemic areas [16, 17]. However, the existing paucity of data on the prevalence of G6PD deficiency impedes the PQ administration in the Republic of Congo. This study aimed to bridge this knowledge gap by investigating the prevalence of G6PD deficiency genotypes in Brazzaville, Republic of Congo. The association between G6PD deficiency with haemoglobin levels and blood cell counts was also assessed.

## Methods

### Study site and sample collection

This study was conducted in Brazzaville, the capital of the Republic of Congo. Malaria transmission is perennial in this area. *Plasmodium falciparum* is the predominant cause of *Plasmodium* species infection [18, 19]. Two hundred and twenty-nine febrile children recruited previously during a previous study conducted at the paediatric hospital Marien NGOUABI between September 2014 and February 2015 were eligible for inclusion in the present study [20]. The inclusion criteria were as follows; participants between one to 10 years old with a fever (≥ 37 °C) or history of fever within 24 h prior to the recruitment in the study. Whole blood samples were collected in vacutainers with EDTA for haemoglobin quantification, malaria diagnosis and molecular analysis. Malaria diagnosis conducted by examining Giemsa-stained thick and thin blood smears using a light microscopy.

### Molecular analysis

#### DNA extraction and *Plasmodium falciparum* detection

The QIAamp DNA blood Mini Kit (Qiagen, Hilden, Germany) was used to extract genomic DNA from archived EDTA blood following the manufacturer’s instructions. DNA samples were stored at − 20 °C. The merozoite surface protein 1 gene (*Pfmsp1*) was used to genotype *P. falciparum* isolates as described elsewhere [21]. This was performed to detect the presence of *P. falciparum* infection.

#### *G6PD* genotyping

A single-step PCR approach was used to amplify a 968 bp fragment of the G6PD gene (*G6PD*) as described by Nguetse et al. [22]. Briefly, DNA was added into a reaction mixture (20 µl) containing 1× PCR buffer, 1× Q-reagent, 1.5 Mm MgCl_2_, 200 µM dNTPs, 100 Nm of each primer and 1U Taq DNA polymerase. The primers (forward primer 5′-GCCCCTGTGACCTCCCGCCA-3′ and reverse primer 5′-GCAACGGCAAGCCTTACATCTGG-3′) used here amplified a *G6PD* fragment containing the 202G>A (rs1050828) and 376A>G (rs1050829) single nucleotide polymorphisms. The thermal cycler conditions were 94 °C for 5 min followed by 30 cycles at 94 °C for 30 s, 67 °C for 1 min and 72 °C for 1 min and a final extension step at 72 °C for 5 min. The amplicons were separated by gel electrophoresis using a 1.2% agarose gel. The PCR products were purified using Sephadex™ G-50 fine DNA grade (GE Healthcare, Buckinghamshire, UK) and subsequently Sanger sequenced by the 3130 xl Genetic Analyzer (Applied Biosystems, CA, USA) in the forward and reverse direction using the foregoing primers and the BigDye Terminator v.1.1 cycle sequencing kit (Applied Biosystems, Foster City, USA). The BioEdit software (http://www.mbio.ncsu.edu/BioEdit/bioedit.html) was used to align the forward and reverse sequences using *G6PD* (NG_009015.2) as a reference, visually inspect the DNA sequence chromatograms to resolve discordant base calls and identify alleles G and/or A at position 202 and alleles A and/or G at position 376 of *G6PD*.

#### Data analysis

The Stata software was used to analyse the data. The Mann–Whitney and Kruskal–Wallis tests were applied to compare the mean ranks between G6PD groups. Chi square test was applied to compare proportions. Significance was observed at a *p*-*value *< 0.05.

## Results

A total of 229 febrile children were eligible for inclusion in this study. Of this, 212 participants were successfully genotyped for the G6PD deficiency. The remaining 4 and 13 participants were excluded from the study due to lack of adequate DNA (degradation of DNA during conservation or no sample available) samples for analyses and failure to obtain nucleotides sequences despite repeat attempts, respectively. The baseline characteristics of the participants are shown in Table 1.

**Table 1:** Baseline characteristics of the participants

Characteristics	Values
Mean age in years ± SD (range)	3.1± 2.5 (1–10)
Sex ratio (M/F)	1:1 (112/100)
*Haemoglobin status*	
Normal (HbAA)	187 (88.2%)
Sickle cell anaemia (HbAS)	25 (11.8%)
Mean haemoglobin level (g/dl)	11.4 ± 4.3
Mean haematocrit (%)	32.8 ± 13.1
Mean red blood cell count (1 × 10^12^/L)	5.1 ± 1.9
Mean white blood cell count (1 × 10^9^/L)	12.4 ± 6.7
Mean platelet count (1 × 10^9^/L)	277.2 ± 158.1
Median parasitaemia (parasites/µl)	10,101 (118.5–280,828)
*Malaria diagnosis*	
Uncomplicated malaria	22 (10.4%)
Submicroscopic infection	52 (24.5%)
Malaria negative	138 (65.1%)

Sanger sequencing was used to determine the G6PD genotype and infer the G6PD phenotype of the participants based on the allele (s) present at nucleotide positions 202G>A (rs1050828) and 376A>G (rs1050829), as described in “Methods” section. Most of the participants (53%; 113/212) had the wild-type G6PD genotype (B hemizygous for males and BB homozygous for females) whereas 13% of the participants had G6PD deficient genotype (A− for males and A−A− for females) (Table 2). The occurrence of G6PD deficient genotype was observed to be significantly higher among male participants (21%) than among the female participants (3%) (*p* < 0.05). In contrast, the presence or absence of *P. falciparum* infection did not associate with G6PD genotype (*p* > 0.05) of male and female febrile children (Table 3). Similarly, the G6PD genotype of male and female Congolese children did not associate with six out of eight study population characteristics (Table 4). The mean red blood and the mean platelet counts were, however, observed to be significantly lower in hemizygous male (G6PD A−) participants than in normal male (G6PD A+ or B) participants (*p* < 0.05). No significant differences were found in the different female G6PD genotype groups.

**Table 2:** The frequency of G6PD genotypes among male and female Congolese children

Gender	Genotype	Genotype Frequency n (%)
Male	B	67 (59.8)
	A+	21 (18.8)
	A−	24 (21.4)
Female	BB	46 (46)
	BA+	22 (22)
	BA−	11 (11)
	A+A+	4 (4)
	A+A−	14 (14)
	A−A−	3 (3)
Overall	*A*− *and A*−*A*−	*27 (13)*

G6PD genotype: male normal = A+ or B; male hemizygous = A−; female normal = BB or BA+ or A+A+; female heterozygous = BA− or A+A−; female homozygous = A−A−; Hb = haemoglobin; Italic = severe G6PD deficiency genotypes

**Table 3:** Distribution of G6PD phenotype among Congolese children based on *Plasmodium falciparum* malaria diagnosis results

Gender	G6PD phenotype	Malaria negative, n (%)	Uncomplicated malaria n (%)	Sub-microscopic infection n (%)
Male	G6PD normal	57 (64.8)	10 (11.4)	21 (23.9)
	G6PD deficiency	19 (79.2)	1 (4.2)	4 (16.7)
Female	G6PD normal	43 (59.7)	8 (11.1)	21 (29.2)
	G6PD heterozygous	18 (72.0)	2 (8.0)	5 (20.0)
	G6PD deficiency	1 (33.3)	1 (33.3)	1 (33.4)

**Table 4:** Association between study population characteristics and G6PD phenotypes in male and female Congolese children

Mean age in years ± SD	3.27 ± 2.51	2.50 ± 1.88	0.106	2.97 ± 2.61)	3.20 ± 2.55	3 ± 2.64	0.843
Parasitaemia geometric mean	4094 (191–151,010)	1000	–	15,486 (158–280,827)	520 (315–862)	73,538	–
Mean haemoglobin level ±SD (g/dl)	11.26 ± 1.83	10.83 ± 0.94	0.118	11.91 ± 7.01	11.06 ± 1.37	10.56 ± 1.25	0.547
*Haemoglobin status*							
HbAA n (%)	78 (88.6)	23 (95.8)	0.296	63 (87.5)	20 (80)	3 (100)	0.507
HbAS n (%)	10 (11.4)	1 (4.2)		9 (12.5)	5 (20)	0 (0)	
Mean haematocrit ± SD (%)	32.48 ± 5.44	30.79 ± 2.94	0.047	34.36 ± 21.43	31.54 ± 4.17	29.76 ± 4.40	0.466
Mean red blood cell count ± SD (10^12^/L0)	5.13 ± 0.80	4.67 ± 0.50	0.001	5.35 ± 3.24	4.8 ± 0.70	4. 62 ± 0.07	0.334
Mean White blood cell count ± SD (10^9^/L)	12.27 ± 5.4	13.29 ± 6.48	0.485	12.9 ± 8.63	11.85 ± 4.67	7.39 ± 2.19	0.247
Mean platelet count ± SD (10^9^/L)	283.27 (149.26)	194.58 ± 141.13	0.01	286.48 ± 166.07	297.08 ± 146.71	369.33 ± 311.18	0.896

Parasite geometric mean given in parasites/µl

## Discussion

This study investigated the prevalence of G6PD deficiency in febrile children from Brazzaville, Republic of Congo. This study shows that 13% of the children were carriers of the severe G6PD deficiency genotype (A− for males and A−A− for females). This finding is consistent with studies conducted in the other Central African countries where the prevalence of G6PD deficiency ranges from 9 to 22% [22–26]. Previous studies reported a higher G6PD deficiency prevalence (22%) in 1988 and 1998 among children with sickle cell anaemia in the current setting [27, 28]. Based on gender, higher G6PD deficiency prevalence was observed among male participants (21%) than female participants (3%). This is consistent with the higher susceptibility of males to G6PD deficiency than females owing to the X-linked inheritance *G6PD* deficient alleles (SNPs) and the hemizygous nature of the X-chromosome in males.

G6PD deficiency has a heterogeneous impact on the severity of malaria. Ruwenda et al. and Nguetse et al. showed that G6PD deficient males and heterozygous females were less susceptible to severe malaria [10, 22]. A similar phenomenon was observed in Cameroon with G6PD deficient participants having reduced parasitaemia, lower severity of anaemia and malaria symptoms and higher haemoglobin compared to the non-deficient individuals [24]. Nevertheless, G6PD deficiency in Kenya and Tanzania was reported to protect only heterozygous females against severe malaria [29, 30], whereas in Mali it protected only hemizygous males against severe malaria [31]. The absence of an association between G6PD deficiency and severe malaria has also been reported previously [12, 32].

A similar heterogeneity has also observed in the protection of G6PD deficiency against uncomplicated malaria. High incidence of uncomplicated malaria was observed in heterozygous females and hemizygous males with G6PD A− compared to participants with the wild-type genotype [33]. Other studies have shown, on one hand, hemizygous males to be more susceptible to uncomplicated malaria whereas others have reported heterozygous females are more susceptible. For instance, Lwanira et al. reported a higher incidence of uncomplicated malaria in Ugandan G6PD deficient males (202G>A) than those carrying the wild-type genotype [34]. Another study from Gabon showed higher uncomplicated malaria incidence in heterozygous females [23]. This finding has also been observed in Ghana as well as in Mali [35, 36]. Even though the study shows that uncomplicated malaria was less prevalent in G6PD deficient males and heterozygous females than in children with G6PD normal, the difference did not reach statistical significance (*p *> 0.05).

Analysis of the association between haemoglobin (Hb) levels and G6PD deficiency in this study showed that mean Hb levels were similar in G6PD deficient children, G6PD heterozygous female children and G6PD normal children. This is congruent with findings from studies conducted in Angola, Ghana and Burkina Faso [25, 35]. Low Hb levels in G6PD deficient patients with sickle cell anaemia (HbSS) [12, 22] and the association of reduced erythrocyte lifespan with G6PD deficiency [37, 38] have also been observed previously.

G6PD deficiency is also an important factor to be considered prior to the administration of some drugs. This is because G6PD is a key enzyme involved in the production of the reduced form of nicotinamide adenine dinucleotide phosphate (NADPH) required by erythrocytes to withstand oxidative stress [2]. Primaquine (PQ) is a classical example of oxidative drugs that induce acute haemolytic anaemia due to the suboptimal activity of G6PD which associated with the presence of *G6PD* SNP(s). PQ is highly efficacious against *P. falciparum* gametocytes [16, 17] and it is recommended, with combination current artemisinin-combined therapy, for the reduction *P. falciparum* transmission [13, 15]. Of this, PQ offers a viable strategy to reduce malaria transmission in the Republic of Congo where malaria is still a major health problem. However, high G6PD deficiency prevalence observed in the present study suggests that caution should be taken before the presumptive use of PQ in the Republic of Congo.

## Conclusions

Taken together, this study provides an update on G6PD deficiency in children from Brazzaville, Republic of Congo. Understanding the distribution of G6PD deficiency in other geographical regions is recommended to inform the use of malaria intervention(s) such as primaquine that induces acute haemolytic anaemia in G6PD deficient individuals.

## References

[1] Peters AL, Van Noorden CJ (2009). Glucose-6-phosphate dehydrogenase deficiency and malaria: cytochemical detection of heterozygous G6PD deficiency in women. J Histochem Cytochem.

[2] Cappellini MD, Fiorelli G (2008). Glucose-6-phosphate dehydrogenase deficiency. Lancet.

[3] Mason PJ, Bautista JM, Gilsanz F (2007). G6PD deficiency: the genotype-phenotype association. Blood Rev.

[4] WHO (2016). Testing for G6PD deficiency for safe use of primaquine in radical cure of *P. viva*x and *P. ovale* malaria. Policy brief.

[5] Vulliamy TJ, Othman A, Town M, Nathwani A, Falusi AG, Mason PJ (1991). Polymorphic sites in the African population detected by sequence analysis of the glucose-6-phosphate dehydrogenase gene outline the evolution of the variants A and A. Proc Natl Acad Sci USA.

[6] Beutler E, Kuhl W, Vives-Corrons JL, Prchal JT (1989). Molecular heterogeneity of glucose-6-phosphate dehydrogenase A. Blood.

[7] Carter N, Pamba A, Duparc S, Waitumbi JN (2011). Frequency of glucose-6-phosphate dehydrogenase deficiency in malaria patients from six African countries enrolled in two randomized anti-malarial clinical trials. Malar J..

[8] Yoshida A, Baur EW, Moutlsky AG (1970). A Philippino glucose-6-phosphate dehydrogenase variant (G6PD Union) with enzyme deficiency and altered substrate specificity. Blood.

[9] WHO Working Group (1989). Glucose-6-phosphate dehydrogenase deficiency. Bull World Health Organ.

[10] Ruwende C, Hill A (1998). Glucose-6-phosphate dehydrogenase deficiency and malaria. J Mol Med (Berl)..

[11] Motulsky AG (1960). Metabolic polymorphisms and the role of infectious diseases in human evolution. Hum Biol.

[12] Clark TG, Fry AE, Auburn S, Campino S, Diakite M, Green A (2009). Allelic heterogeneity of G6PD deficiency in West Africa and severe malaria susceptibility. Eur J Hum Genet.

[13] WHO (2015). WHO policy brief on single-dose primaquine as a gametocytocide in *Plasmodium falciparum* malaria.

[14] Beutler E (1994). G6PD deficiency. Blood.

[15] WHO (2015). Guidelines for the treatment of malaria.

[16] Graves PM, Choi L, Gelband H, Garner P (2018). Primaquine or other 8-aminoquinolines for reducing *Plasmodium falciparum* transmission. Cochrane Database Syst Rev.

[17] Lin JT, Lon C, Spring MD, Sok S, Chann S, Ittiverakul M (2017). Single dose primaquine to reduce gametocyte carriage and *Plasmodium falciparum* transmission in Cambodia: an open-label randomized trial. PLoS ONE.

[18] Trape JF, Peelman P, Morault-Peelman B (1985). Criteria for diagnosing clinical malaria among a semi-immune population exposed to intense and perennial transmission. Trans R Soc Trop Med Hyg.

[19] Trape JF, Zoulani A (1987). Malaria and urbanization in central Africa: the example of Brazzaville. Part II: results of entomological surveys and epidemiological analysis. Trans R Soc Trop Med Hyg.

[20] Etoka-Beka MK, Ntoumi F, Kombo M, Deibert J, Poulain P, Vouvoungui C (2016). *Plasmodium falciparum* infection in febrile Congolese children: prevalence of clinical malaria 10 years after introduction of artemisinin-combination therapies. Trop Med Int Health..

[21] Gueye NSG, Ntoumi F, Vouvoungui C, Kobawila SC, NKombo M, Mouanga AM (2018). *Plasmodium falciparum* merozoite protein-1 genetic diversity and multiplicity of infection in isolates from Congolese children consulting in a pediatric hospital in Brazzaville. Acta Trop.

[22] Nguetse CN, Meyer CG, Adegnika AA, Agbenyega T, Ogutu BR, Kremsner PG (2016). Glucose-6-phosphate dehydrogenase deficiency and reduced haemoglobin levels in African children with severe malaria. Malar J..

[23] Migot-Nabias F, Mombo LE, Luty AJ, Dubois B, Nabias R, Bisseye C (2000). Human genetic factors related to susceptibility to mild malaria in Gabon. Genes Immun.

[24] Awah FM, Uzoegwu PN (2008). Malaria protection in glucose-6-phosphate dehydrogenase deficient individuals in Bamenda population of Cameroon. Glob J Pure Applied Sci..

[25] Brito M, Tchonhi CL, Santos B, Veiga L (2014). Glucose-6-phosphate dehydrogenase deficiency in children from 0 to 14 years hospitalized at the Pediatric Hospital David Bernardino, Luanda, Angola. J Pharmacogenomics Pharmacoproteomics..

[26] Barker MK, Henderson AM, Naguib K, Vercauteren SM, Devlin AM, Albert AY (2017). Serum soluble transferrin receptor concentrations are elevated in congolese children with glucose-6-phosphate dehydrogenase variants, but not sickle cell variants or alpha-thalassemia. J Nutr.

[27] Andoka G, Thiloemba A, Moussoki J, Djembo-Taty M, Galacteros F (1988). [Evaluation of the incidence of glucose-6-phosphate dehydrogenase deficiency in children with sickle cell anemia in Brazzaville (Congo)]. Med Trop.

[28] Bouanga JC, Mouele R, Prehu C, Wajcman H, Feingold J, Galacteros F (1998). Glucose-6-phosphate dehydrogenase deficiency and homozygous sickle cell disease in Congo. Hum Hered.

[29] Manjurano A, Sepulveda N, Nadjm B, Mtove G, Wangai H, Maxwell C (2015). African glucose-6-phosphate dehydrogenase alleles associated with protection from severe malaria in heterozygous females in Tanzania. PLoS Genet.

[30] Uyoga S, Ndila CM, Macharia AW, Nyutu G, Shah S, Peshu N (2015). Glucose-6-phosphate dehydrogenase deficiency and the risk of malaria and other diseases in children in Kenya: a case-control and a cohort study. Lancet Haematol..

[31] Guindo A, Fairhurst RM, Doumbo OK, Wellems TE, Diallo DA (2007). X-linked G6PD deficiency protects hemizygous males but not heterozygous females against severe malaria. PLoS Med..

[32] Lell B, May J, Schmidt-Ott RJ, Lehman LG, Luckner D, Greve B (1999). The role of red blood cell polymorphisms in resistance and susceptibility to malaria. Clin Infect Dis.

[33] Parikh S, Dorsey G, Rosenthal PJ (2004). Host polymorphisms and the incidence of malaria in Ugandan children. Am J Trop Med Hyg.

[34] Lwanira CN, Kironde F, Kaddumukasa M, Swedberg G (2017). Prevalence of polymorphisms in glucose-6-phosphate dehydrogenase, sickle haemoglobin and nitric oxide synthase genes and their relationship with incidence of uncomplicated malaria in Iganga, Uganda. Malar J..

[35] Amoako N, Asante KP, Adjei G, Awandare GA, Bimi L, Owusu-Agyei S (2014). Associations between red cell polymorphisms and *Plasmodium falciparum* infection in the middle belt of Ghana. PLoS ONE.

[36] Maiga B, Dolo A, Campino S, Sepulveda N, Corran P, Rockett KA (2014). Glucose-6-phosphate dehydrogenase polymorphisms and susceptibility to mild malaria in Dogon and Fulani. Mali. Malar J..

[37] Patel H, Savalani P, Dhanani H, Patel RZ, Joshi SR (2016). Red cell parameters in G6PD deficient individuals during normal steady state. J Med Sci Clin Res.

[38] Sanna G, Frau F, Melis MA, Galanello R, De Virgiliis S, Cao A (1980). Interaction between the glucose-6-phosphate dehydrogenase deficiency and thalassaemia genes at phenotype level. Br J Haematol.

